# Dr. Mackenzie on the Natural Cure of Diseases

**Published:** 1847-04

**Authors:** William Mackenzie


### PART THIRD.

### CONTRIBUTIONS TOWARDS THE ADVANCEMENT OF THE Natural History and Treatment of Diseases.

### II. A SKETCH OF THE NATURAL CURE OF DISEASES.

BY WILLIAM MACKENZIE, M.D.

[The following paper was long since published in the Glasgow Medical Journal (No. v, Feb. 1829) of which the accomplished writer was then Editor. It bears so appropriately on the general subject to which this part of our journal is for the present devoted, that we make no apology for reprinting it. We should be under still greater obligation to the author, if he would favour us with new and still more extensive illustrations of the important practical principles advocated by him.]

As the greater number of diseases which attack the human frame are susceptible of cure by the operations of Nature alone, while no one disease can be cured by the powers of Art alone, and as in all our attempts to cure diseases by artificial means, we imitate, or ought to imitate, the modes of cure followed by Nature, there is perhaps no question more truly of importance to medical practitioners than this :—What are the natural processes by which diseases are removed?

Before attempting to answer this question, it may not be improper to premise, that in speaking of the restorative power, or powers of Nature, and of the natural cure of diseases, I have no intention to admit the existence of any intelligent power or powers resident in the body, superintending or operating in the cure. The *vis conservatrix* and *vis medicatrix Naturæ* have sometimes been spoken of in such a manner as might lead us to suppose that this was actually meant—that it was not merely acknowledged that the body has in itself a power or condition, depending on its structure and on the revolutions of its functions, by which, in many cases, it resists the injuries which threaten it, and on many occasions corrects or removes the disorders induced in it, but that this preserving and remedying power was an actual agent superadded to the constitution of the body.

It will at once be seen that I refer in these remarks to the opinions of the celebrated Stahl, who explicitly founded his system on the supposition, that the power of Nature, so much talked of, did not in any degree depend on the structure and functions of the body, but belonged entirely to what he styled the *Rational Soul.* He maintained that, on many occasions, the soul acts even independently of the state of the body; and that without any physical necessity arising from that state, but purely in consequence of its intelligence, the soul perceiving the tendency of noxious powers threatening, or of disorders anywise arising in the system, immediately excites such actions in the body as are suited to obviate the pernicious consequences which might otherwise take place. This is a very fanciful, and I believe an entirely groundless hypothesis; but there is so much appearance of intelligence and design in the operations of the animal economy, that many medical authors have very much countenanced the same opinion. It may suffice at present to observe upon these notions, that the admitting of any such intelligent governor of the animal economy, would oblige us to reject all anatomical and physiological reasoning concerning diseases, and would render the whole practice of medicine capricious, and even dangerous. We see, in fact, the preposterous effects of such a system in the practice of Stahl himself and his followers, who, trusting much to the constant attention and wisdom of nature, proposed what they called the art of curing diseases *with expectation*, used, therefore, only very inert and frivolous remedies, were extremely reserved in the use of such general and powerful means of cure, as bloodletting, vomiting, and the like, zealously opposed the use of some of the most efficacious medicines, such as opium and Peruvian bark, and, in fact, converted the healing art, as far as they could, into a mere curious contemplation of diseases and of death.

I go on, then, in answer to the question already stated, to remark in the first place, that in some instances the natural cure of diseases is so direct and prompt, that we are unable to discover any process by which it is effected. Pain ceases to be felt—and the disease is at an end. Spasm relaxes—and the muscle returns under the control of volition. In epilepsy, we see the return to health take place almost as suddenly as was the attack of the disease;—in the midst of the most violent symptoms, that calm comes on which announces the termination of the attack. The heart interrupts or relaxes its wonted action, and as the common name of the disease denotes (ςυν κοπτω,) the patient is struck down, blind, pale, and cold;—the heart resumes its labour, and animation is restored. In cases such as these, the cure is so simple, the steps of recovery so few and short, that we may be allowed to regard them as instances of the mere cessation of disease

In the second place, some diseases undergo a natural cure by means of the *Revolutions of the Functions.* As an example of this, we may take the disease of intoxication. When a person has swallowed a large quantity of ardent spirits, there follow all the symptoms produced by a narcotic poison. For a time the force of the circulation is increased, but this is soon followed by languor, delirium, and stupor, attended by nausea, vomiting, and headache. A tendency to apoplexy is produced, and a temporary want of power over some of the voluntary, and occasionally in some of the involuntary movements of the body. In consequence of this palsy extending to the muscles of respiration, the disease of intoxication sometimes proves mortal; but in general a cure is effected by the natural revolutions of the functions of the body. We are unable to trace the processes completely by which this disease is produced and afterwards cured, but part of them is sufficiently known. It is known that the ardent spirits are carried into the circulation, for alcohol has been obtained by distillation from the blood of animals to whom it had been administered. It is also known, that mixed with the blood, it first of all excites the heart to more frequent action; and either directly, in the blood circulating through the brain, or indirectly, by operating on the nerves of the stomach, excites the brain to inordinate action, and to all the false fire and unmeaning fury of the drunkard. This is followed by congestion of the blood passing through the vessels of the brain, and this is succeeded by temporary palsy and insensibility. The method in which the body gets free of the poison is partly by its being conveyed in the blood to the kidneys, by which organs it is separated along with the urine, and thus excreted, and partly by being conveyed to the perspiring organs, namely, the skin and the lining membrane of the lungs, so that mixed with their secretions, the sweat and the vapour of the breath, it is thus let free from the blood, and consequently from the body, which it had so much deranged.

This is the method in which Nature relieves herself of this disease, and of the poison by which it was occasioned; and it is probable that in this very way she relieves herself of other poisons, and of still more formidable and tedious diseases. We are unable, it is true, to detect the poison of fever; but we have scarcely a doubt of its existence, of its absorption by the lining membrane of the lungs, of its circulation through the body in the blood, exciting languor, weakness, pain, rapid motion of the heart, disorder of the brain, oppression of all the functions, and frequently death, while in favorable cases, we observe, that by augmented secretion of urine, or increased flow of perspiration, the disease is terminated, and the poison probably expelled from the system. It is this natural cure of fever which the medical attendant hails with so much gladness. It is for this he watches—and returning again and again to the bedside of his patient, feels the skin, and looks at the eye, and examines the tongue. If he discovers the least kindly moisture exuding from the surface of the body, he hails it as the signal of life, and welcomes it with a heart as happy as that with which the alchemist would have welcomed a drop of gold which he had long anxiously looked for, and almost despaired to see.

And in the cure of fever we but imitate this natural cure. We adopt a cautious and symptomatic treatment, and endeavour rather to assist the slow proceedings by which Nature frees herself of the disease, than to force it to retire. In the treatment of fever, no doubt, some have attempted to set up new actions in the constitution, by which to suspend, or even wholly to remove, the disease. But when we find that in almost every case of this disease, the vital powers are of themselves sufficient to effect the most perfect recovery, ought we not rather to adopt the indirect method of cure, watching the curative operations of Nature, supporting and aiding her in these operations, setting aside impediments, that the vital powers may exert their beneficial influence in a free and undisturbed manner, removing or alleviating dangerous individual symptoms, until the disease be surmounted, and the curative process completely and happily terminated? I have had opportunities of witnessing both plans of treating fever—the direct, in which an attempt is made by active treatment to dispel the disease—and the indirect, in which the natural cure is imitated—and I have no hesitation in saying, that though the former might serve more to flatter the self-complacency of the practitioner, the latter was by far the more successful.

The body is almost altogether fluid. Nine-tenths of its weight are so, and only one-tenth solid. The fluid parts are in a perpetual state of change, being decomposed by one set of functions, and recomposed by another; and although it may be doubted how far these changes extend to the solid fibres or parenchyma of the body, it is unquestionable that our fluids, by means of digestion, absorption, circulation, respiration, and secretion, are in constant revolution. By these processes there is effected an uninterrupted decay and restoration of the body; and we cannot doubt, that the natural cure of diseases depends very much on the existence and on the perfection of this revolution. Nay, it is extremely probable that one of the principal intentions served in this mode of carrying on life, is the prevention and removal of diseases. It is also chiefly by substances entering into the course of this same system of circulations and of changes, going through the absorbents into the blood, with the blood passing through all parts of the body, and coming at last to the kidney and to the skin, that we are able to imitate those cures of diseases which are the most perfectly effected by the restorative powers of Nature.

In the third place, the natural cure of diseases appears, in some instances, to be governed by *Revolutions of Time.*

That some diseases are periodic—that they come, and go, and return, and are again removed, at regular intervals of time, admits of no manner of doubt. It is also indubitable that the healthy actions of the body are, in a certain degree, associated with periodic revolutions. The periods of sleep and of menstruation are striking examples. The ordinary repletions of the body, and the ordinary evacuations are also periodic. The instincts of man, though less under the control of seasons than those of the inferior animals, are called only into periodic activity. Even the noblest powers of the mind, in the noblest of the species, as in Milton, are subject to periodical returns of exhaustion and of force. This periodical tendency necessarily attends the morbid actions as well as the healthy actions of man. Rheumatism, fevers of different kinds, lunacy, gout, and several other diseases, manifest their periods of return and their periods of departure in the most unquestionable manner. Whether or not these phenomena of health and of disease actually arise from the diurnal, lunar, and annual mutations of the earth, and its satellite, affecting the living body, I shall not pretend to decide.

In ancient times, the heavenly bodies, and especially the sun and moon, were supposed to influence all diseases, but particularly mania and epilepsy. When the Newtonian doctrine of universal attraction was first promulgated, Dr. Mead revived and supported the ancient doctrine with great learning and ingenuity. It was regarded merely as an ingenious conjecture and possible fact, till Dr. Darwin, by interweaving it with his peculiar doctrines, once more endeavoured to give it an air of serious importance. Dr. Balfour brought it forward as capable of direct proof. His opinion is, that the influence of the sun and moon, when in a state of conjunction, which is named sol-lunar influence, produces paroxysms in continued fever, in all cases in which a paroxysmal diathesis exists; and as this influence declines, in consequence of the gradual separation of these luminaries from each other, and their getting into a state of opposition, a way is left open to the system for a critical and beneficial change. Dr. Stoker put Dr. Balfour's doctrines to the test of 276 patients, between July 6th and September 6th, 1817, in Dublin. He has given us his tables, and observes, that "very little coincidence indeed is to be remarked from a view of these tables." *

That there is nothing impossible in sol-lunar influence—that the attraction of the sun and moon, which raises the ocean into mountains, may also affect the nice sensibilities of animal bodies—and that medical meteorology, more carefully studied, may yet come to unfold the origin, and trace the apparently capricious courses of many diseases, we may readily admit. Indeed this science, including, as it does, the effects of changes in temperature and humidity, as well as of seasons, on the human constitution, must be regarded as yet in its infancy, and as affording more scope for original observations and inquiries than any other branch of medicine.

Even, however, in the present state of our knowledge, the periodic departure of some diseases is a fact of high importance to the medical practitioner. It not merely enables him to pronounce a more accurate prognosis than if he were ignorant of the fact; but he seizes with advantage the period of intermission for the exhibition of the remedies which are found to operate against the tendency which all periodic diseases have to return.

In the fourth place, as Mr. Hunter has given to interstitial and progressive absorption, the title of the Natural Surgeon, and as there are various other processes of an analogous kind, materially concerned in the cure of diseases by nature, I shall include them all under the name of the processes of Natural

* See Good's Study of Medicine, vol. ii, p. 90. Second edition. London, 1825.

Surgery. They distinctly differ from the processes of health; and are so remarkably useful in the preservation of life, as to have deeply attracted the attention of medical philosophers. The most important of them are—1st, the closure of divided blood-vessels; 2d, adhesion, or union by the first intention; 3d, granulation, and union by the second intention, including the union of fractured bones, and cicatrization; and, 4th, interstitial, progressive, and ulcerative absorption.

The means employed by Nature in stopping hæmorrhagy from wounded blood-vessels, are, the retraction of the vessel, if completely cut across; the contraction of its open extremities; the formation of a coagulum within the vessel; and the injection of the surrounding cellular substance with blood, and the coagulation of that blood. I need not say how minutely these different particulars in the natural cure of hæmorrhagy have been studied; nor how ingeniously and successfully they have been imitated and improved upon, in the artificial means adopted for the closure of wounded blood-vessels. The natural cure of internal hæmorrhagies is accomplished by means analogous to those by which external hæmorrhagies are arrested.

When a bone is fractured, a muscle torn across, a tendon ruptured, or an incision made into any part, steps are immediately taken by Nature for reparation. When the incision is made by a clean cutting instrument, and the sides of the wound are not widely separated, the union is more directly and speedily effected; but even then, the means are essentially the same as in union by what is termed the second intention, or in the healing of fractured bones, torn muscles, or ruptured tendons. A quantity of blood is extravasated into the line of separation between the divided parts, whatever these may be, and into the cellular membrane around the injured parts; when the blood effused is not very considerable in quantity, and the parts are not much injured, the parts which had been divided gradually approach each other. In a few hours, the surfaces of the fracture, laceration, or wound, are covered with a layer of fibrin. If the two divided surfaces have been kept in contact, the fibrin is found adhering to both, and serving as a slight bond of union between them. That this fibrin intervening between the surfaces of a wound, fracture, or laceration, is derived partly from the vessels divided by the injury, can scarcely, I think, be doubted, although its origin has more commonly been supposed to be the capillaries situated close to the divided surfaces. The next step in the process of reparation is the organization of the effused fibrin. It becomes penetrated by arteries, veins, and absorbents, shooting from the vessels of the neighbouring parts. The completion of this part of the process is the completion of adhesion, if the sides of the wound had luckily remained or been brought and kept in contact; but if this had not been the case, the organized fibrin assumes the appearance of red fleshy points or granulations, while a secretion of pus flows from the exposed surface, and the union is completed by gradual approximation and contraction. In the case of fractured bones, the effused fibrin first of all becomes cartilaginous, and then osseous.

That variety of absorption by which a part, or the whole of the body is wasted, has been called by Mr. Hunter interstitial; because it is removing parts of the body out of the interstices of that part which remains, leaving the part still as a perfect whole; although, in some cases, it must be confessed, it is carried on till not a vestige of the part remains. Progressive absorption, again, is that process by which pus, and extraneous bodies of all kinds, are brought to the external surface. Ulcerative absorption may be regarded as a substitute for mortification; and it seems to take the place of this loss of all action, from a degree of vigour superior to that which exists in the parts where mortification takes place. These modes of absorption are often united, or succeed each other. Although the last of them would often appear to be doing mischief, by destroying parts which are of service, yet in all cases we may refer consecutive absorption to some necessary purpose. We may depend upon it, as Mr. Hunter observes, that those parts have not the power of maintaining their ground, and that it becomes a substitute for mortification.

As for progressive absorption, Nature has not only made what might be called an instinctive provision in the parts to remove themselves, so as to bring extraneous bodies to the skin for their exit, and thereby guarded the deeper seated parts, but has also guarded all passages or outlets, into which we might perhaps suppose, though extraneous bodies were discharged, no great mischief could follow. Thus, an abscess in the cheek, close on the internal membrane of the mouth, and some way from the skin, shall not, as we might perhaps have thought it should, open into the mouth, but shall push outwards, and at last come to point and break externally.

Cicatrization is a process of Natural Surgery, in which there is invariably betrayed a great degree of economy, for we never find the new formed skin so large as the sore was on which it is formed. Indeed, when the looseness of the surrounding skin permits it, almost no new skin is formed, as in the scrotum.

In the fifth place, Nature often cures or prevents one disease by producing another; or, as this process has been termed, by *converting one disease into another;* and this is a plan, which, in the practice of medicine, we frequently endeavour to imitate, exactly as we attempt to imitate also the complete removal of diseases which Nature accomplishes in other cases.

Diarrhœa is a disease, and yet it is a means not unfrequently adopted by Nature for the cure of dropsy; and we often imitate this cure, by giving hydragogues. If a hæmorrhoidal discharge which had continued to flow for a number of years at accustomed periods should cease, the individual is apt to become affected with giddiness, pain in the head, and a threatening of apoplexy; but if the discharge of blood from the rectum returns, these symptoms of impending apoplexy are removed; and if the discharge does not of itself return, we often apply leeches round the anus with the best effects. These are instances of *related diseases,* or of the cure of diseases by *conversion;* the dropsy being converted into a diarrhœa, the apoplexy into a discharge of blood from the rectum.

It would appear that there are two varieties of conversion. The one may be called *sanative*, for it produces health; and the other *insanative,* for though it removes one disease, it does not restore health.

For instance, a patient with headache is seized with spontaneous epistaxis, by this means the natural state of circulation within the head is restored, the headache is removed, the epistaxis ceases, and health is completely recovered. This I should call a sanative conversion.

Again, I once saw a man with diabetes become suddenly affected with dropsy. He had œdema, an evident collection of water in the cavity of the peritoneum, the symptoms of hydrothorax, and even those of hydrocephalus. In fact, I never saw so universal a case of dropsy. As soon as the dropsical symptoms made their appearance, the diabetic symptoms entirely ceased, the urine was reduced to an ordinary, or even less than ordinary quantity, and lost entirely its sweet taste. I should call this an insanative conversion. The change—the cure of the diabetes—was not accompanied by any tendency to health. By means of diuretic medicines, the dropsy was removed, and that instant the diabetes returned. After some time, the diabetes again suddenly stopped, and the dropsy returned. I am not certain but that this morbific conversion was repeated thrice, till at length the man died, his constitution being completely worn out by these alternating diseases.

Dr. Parry, in the first volume of his 'Elements of Pathology and Therapeutics,' has related a number of striking cases illustrative of this subject. Of these I shall select a few.

Headache, depression of spirits, and other nervous affections with which a young lady had been long afflicted, were completely cured by the measles.

In a gentleman labouring under gout, the fit was immediately removed by an accidental catarrh from cold.

In a lady, chronic rheumatism of the right shoulder was immediately and permanently relieved on the appearance of jaundice, with pain, from a gall-stone, which did not pass.

A headache, of some years' duration, subsiding, was followed by a cough, accompanied with incessant and wasting hectic fever. After the man had been long confined to his bed, and death was every day expected, the headache began slightly to return; and as it became established, the cough and fever receded, and the patient regained his flesh, but continued subject to headache as before.

In a lady, various nervous symptoms disappeared on the commencement and gradual increase of a vascular fulness of one mamma. The progress of this disease was suspended in its turn by rheumatism in the hip; and again, the rheumatism disappearing, the affection of the mamma rapidly increased.

In a gentleman, long accustomed to violent vertigo and pain in the head, these affections were constantly relieved on the coming on of œdematous swellings in the legs and feet.

In a lady, mania, which ended in suicide, alternated with œdematous swelling of the ankles.

In a gentleman, epileptic fits, which used to occur at least once a-week, were suspended for three weeks by pneumonia; but returned with additional frequency after the pneumonia had ceased.

Bronchocele, in a lady, gradually disappeared during the progress of a fatal inflammation of the liver.

In a young man, long continued cough, accompanied with fever, night-sweats, and emaciation, ceased on the spontaneous occurrence of inflammation and ulceration under the scapula.

From having noticed, agreeably with the observations of physicians and of the vulgar in all ages, that the occurrence of certain diseases is preventive of some and curative of others, one author, Sir George Smith Gibbes, of Bath, has attempted to generalize this propensity in nature. He has concluded, as disease sometimes appears to be curative of disease, that one disease is always necessary to the cure of another, that just as many functions undergo a secondary derangement as are necessary for the cure of the primary one, and that no diseases occur but such as are curative in their effects or in their tendency.

The work in which this doctrine is maintained I have not had an opportunity of perusing; but an abstract and refutation of Sir G. S. Gibbes' opinions are given by Dr. Pring in his 'Exposition of the Principles of Pathology.' Notwithstanding his general condemnation of the doctrine, Dr. Pring allows that some good has arisen from this attempt at systematizing; observing that men, from attachment to their systems, became zealously attentive to everything which can reflect credit on their opinions, and not unfrequently, by showing phenomena in a new or more conspicuous light, serve the cause of science, although they fail in their immediate object. Sir G. S. Gibbes is fully entitled to the benefit of this remark. From observing particularly the curative tendency of diseases, he has indicated the propriety of not resisting secondary diseases in many instances, and of imitating them, when they tend to be curative, in many others.

Such, then, is a very short and imperfect sketch of the modes or processes by which Nature effects the removal of diseases. I have touched upon each, more for the purpose of recalling those interesting facts to the recollection of my readers, than with the hope of laying before them a single idea which can be new to them. I may perhaps be permitted to say, however, that I apprehend the natural cure of diseases to be a matter too little studied by us all, and especially too much neglected by those whose business it is to introduce beginners to the profession of the healing art. In fact, too much attention is bestowed, in the first instance, on what Art, and too little on what Nature can do; whereas, the "*quid natura faciat, aut ferat,*" ought first to be well understood, and then the attention turned to the subsidiary contrivances of medicine. As our profession is now studied, interference—perpetual and busy interference—is too much encouraged; observation and caution too much neglected; nor is it sufficiently impressed on the minds of those who mean to practise medicine, that the whole of the Healing Art is but an imitation of the different modes of Natural Cure, and that in general our cures are cures by conversion,—in other words, that we alleviate or remove one disease, in most instances, only by means of another which we excite, and very rarely effect anything like direct relief. I know that this view of the Healing Art is not a flattering one; but there can be no doubt that it is a true, and might be, a useful one. A rational Theory of Medicine can be built only on the Natural Cure of Diseases.

Glasgow, 24th September, 1828.

---

